# Simultaneous Nasopharyngeal Carriage of Two Pneumococcal Multilocus Sequence Types with a Serotype 3 Phenotype

**DOI:** 10.1155/2010/765479

**Published:** 2010-12-01

**Authors:** Donald Inverarity, Mathew Diggle, Roisin Ure, Diego Santana-Hernandez, Peter Altstadt, Timothy Mitchell, Giles Edwards

**Affiliations:** ^1^Monkland Hospital, Clinical Microbiology Department, Monklands General Hospital, Monkscourt Avenue, Airdrie, Lanarkshire ML5 0JS, UK; ^2^Queens Medical Centre, Department of Clinical Microbiology, Nottingham University Hospitals NHS Trust, Derby Road, Nottingham NG7 2UH, UK; ^3^Scottish Haemophilus, Legionella, Meningococcal and Pneumococcal Reference Laboratory (SHLMPRL), Stobhill Hospital, Glasgow G21 3UW, UK; ^4^Fundación Totaí, Casilla 158, Trinidad, Beni, Bolivia; ^5^Laboratorios Altstadt, Casilla 158, Trinidad, Beni, Bolivia; ^6^Institute of Infection, Immunity and Inflammation, Glasgow Biomedical Research Centre University of Glasgow, College of Medical, Veterinary and Life Sciences, 120 University Place, Glasgow G12 8TA, UK

## Abstract

Knowledge of the epidemiology of pneumococcal disease in Bolivia is sparse, and Multilocus Sequence Typing (MLST) of isolates has not been previously possible. Beni state has until recently been a geographically isolated region of the Bolivian Amazon basin and is a region of significant poverty. During June and July 2007, we performed a pneumococcal carriage study recruiting over 600 schoolchildren in two towns in the Beni state. Here, we describe the unique identification of simultaneous nasopharyngeal carriage of two pneumococcal multilocus sequence types with a serotype 3 phenotype within a single subject.

## 1. Introduction

Multilocus sequence typing (MLST) is an internationally utilized method for the molecular categorization of *Streptococcus pneumoniae *(the pneumococcus) [1]. Pneumococci predominantly colonise the human nasopharynx and in the vast majority of instances do not progress to cause invasive disease. In the first two years of life, 95% of children can be colonized with pneumococci and 73% can acquire at least two different serotypes, although these are carried on different occasions. Data relating to multiple colonisation is limited; however, the range of multiple colonisation when studied can vary dramatically from 1.3% to 30%. It is important to note that a number of different factors could influence this, including, geographical locations, social and economic factors, and sample technique [2–4]. Nasopharyngeal colonization can begin as early as the day of birth. The duration of carriage for a particular serotype is commonly 2.5 to 4.5 months, and the duration of carriage decreases with each successive pneumococcal serotype. This duration of carriage is inversely correlated with age [4] as pneumococcal carriage declines as children grow older [5]. Although it is well documented that multiple different serotypes (and consequently multiple sequence types) of pneumococci may be carried in the nasopharynx concurrently, we are unaware of any descriptions of multiple sequence types of the same serotype being identified simultaneously at this site. 

As part of a study of pneumococcal carriage among schoolchildren from the Beni region of Bolivia during June and July 2007, we performed a pneumococcal carriage study recruiting over 600 schoolchildren in two towns in the Beni state. Here, we describe the unique identification of simultaneous nasopharyngeal carriage of two pneumococcal multilocus sequence types with a serotype 3 phenotype within a single subject. 

## 2. Materials and Methods

This study was designed in accordance with the standard method of the WHO working group [6] and an earlier method devised by PAHO for a Latin American context http://www.paho.org/Spanish/ad/ths/ev/LABS-manual-vigilancia-serotipos.pdf {accessed 10th of October 2008}. Dacron polyester-tipped swabs (Medical Wire and Equipment, UK) were couriered from the United Kingdom for nasopharyngeal swabbing as were Skim Milk Tryptone Glucose Glycerin (STGG) broth media [7] which had been manufactured, sterilized, and quality controlled as 1 ml aliquots at the Scottish Haemophilus, Legionella, Meningococcal, and Pneumococcal Reference Laboratory (SHLMPRL) in cryotubes (Sarstedt AG & Co., Germany) to be used as a short-term transport media and storage media at −20°C. 

Five percent horse blood agar (E & O Media Services Limited, United Kingdom) was couriered from the United Kingdom as were optochin discs (Oxoid, United Kingdom) and Transwabs (TSCswabs, United Kingdom). The use of 5% horse blood rather than blood agar with gentamicin [8], colistin-nalidixic acid, or colistin-oxolinic acid was a necessary deviation from the published standard method [6]. 

Nasopharyngeal swabs were taken by an experienced otolaryngologist (Dr. Santana-Hernandez). If nasopharyngeal swabbing was not tolerated or not possible in younger children, oropharyngeal swabs were performed. The tips of the swabs were then cut off and stored in STGG and either plated onto 5% horse blood agar on the same day or stored at −20°C until cultured. After incubation, alpha haemolytic colonies were subcultured onto 5% horse blood agar for optochin susceptibility testing. Incubation was performed at 37°C in a carbon dioxide-enriched atmosphere using candle jars at Laboratorios Altstadt, Trinidad, Bolivia. 

Pure cultures of presumed pneumococci were stored at room temperature on Transwabs (TSCswabs, United Kingdom) until ready for transportation to SHLMPRL by air [9]. 

Facilities for serotyping in Latin America are sparse [10], and it is not possible to perform MLST in Bolivia. Transportation of isolates from Trinidad, Bolivia to Glasgow, United Kingdom took 42 days on Transwabs under conditions which were not environmentally controlled.

Blood agar with neomycin (Oxoid, United Kingdom) was used at SHLMPRL to culture isolates received on Transwabs for 48 hours under anaerobic conditions. Isolates which had survived transportation were further subcultured on 5% horse blood agar and stored at −80° on Protect beads (TSC Ltd, United Kingdom). 

The whole process of serotyping of strains was performed at SHLMPRL using a coagglutination method [11] utilising sera from Statens Serum Institut, Denmark.

MLST was performed on these isolates as described previously [12–14]. Briefly, fragments from the seven housekeeping genes, *aroE*, *gdh*, *gki*, *recP*, *spi*, *xpt, *and *ddl* were amplified from the pneumococcal lysate with the primers described by Enright and Spratt [13] by using a single PCR reaction. The amplified DNA was cleaned as previously described [12, 15]. The cleaned amplified DNA was then sequenced with the same primer set using the DYEnamic ET Terminator sequencing kit (Amersham Biosciences, Little Chalfont, United Kingdom). The subsequent sequenced DNA was cleaned as previously described [12, 15]. These procedures were carried out on a liquid handling robotic platform (MWG-Biotech, Milton Keynes, United Kingdom) and a MegaBACE 1000 DNA sequencer (Amersham Biosciences). The analysis of the sequence data and the subsequent assignment of a sequence type (ST) were performed as described previously [16]. Further analysis of relationships between this and other pneumococcal STs was performed using the BURST (Based Upon Related Sequence Types) program [17].

## 3. Results

This pneumococcal carriage study was conducted among schoolchildren from the Beni region of Bolivia, and during this study we identified a 9-year-old girl with mucoid pneumococci present in the nasopharynx. Colonies of this single mucoid phenotype consistently were identified as serotype 3 using a coagglutination method [11]. MLST was performed on two separate colonies which were indistinguishable morphologically and identified one as Sequence Type 180 (ST180) and the other as ST1989. These sequence types differ by two of the seven housekeeping genes used in the MLST scheme. Sequence Type 1989 (ST1989) and ST180 exist as double-locus variants within the same clonal complex. ST1989 may possibly have arisen from ST2311 which may have arisen from ST180. The single-nucleotide polymorphisms (SNPs) in the *xpt* and *gdh* alleles account for these differences in sequence type.

## 4. Discussion

Serotype 3 pneumococci are morphologically distinct from most other serotypes of pneumococci due to their mucoid capsule [18]. It has been determined that duplications in the *cap3A* gene in the type 3 capsule locus are associated with high-frequency phase variation [19] which relates to capsular and acapsular (rough) phase variants [20]. 

This mucoid serotype is also a common cause of acute otitis media [20, 21], particularly ST180 [22], where bio-film formation may be important in the pathogenesis of this manifestation. Also, Serotype 3 pneumococci cause acute conjunctivitis, and it is postulated that this serotype possesses virulence factors which predispose it to mucosal sites [21]. In addition, Serotype 3 is associated with an increased relative risk of death from invasive pneumococcal disease in Swedish adults [23], but in children, Serotype 3 ST180 pneumococci have been identified as having odds of invasiveness of only 0.1 which was significantly associated with asymptomatic carriage [24]. Due to the limited data, the odds ratio of the newly identified ST-1989 is unknown. The dichotomy that Serotype 3 pneumococci can cause disease with a high associated mortality in some individuals while being harmlessly carried in the nasopharynx of others has been recognised since the early 20th century [25]. An association between Serotype 3 pneumococci causing disease more commonly in the elderly than in children is also an established observation [25]. Serotype 3 isolates of different genotypes may also have different virulence in mice [26, 27]. 

We believe that this discovery of a population of double locus variants expressing the Serotype 3 capsules concurrently in a human host is suggestive of a number of different events. It is possible that multiple spontaneous mutations resulting in single nucleotide polymorphisms occur naturally within nasopharyngeal populations of pneumococci which, in a natural biofilm environment, may be contributing to genetic diversity and genetic exchange *in vivo. *This could result in altered interactions between the Serotype 3 pneumococcal populations and their hosts depending on which genotype was predominant, and it is possible that this might influence disease manifestation and outcome. Moreover, it is possible that these variants diverged from a single genetic source long before acquisition by this host and subsequent isolation and characterization. Therefore, although these findings are valuable within the context of such a unique geographic location and social and economic environment, this data is limited and further follow-up studies would be required to support any of these findings. 

## Figures and Tables

**Figure 1:**
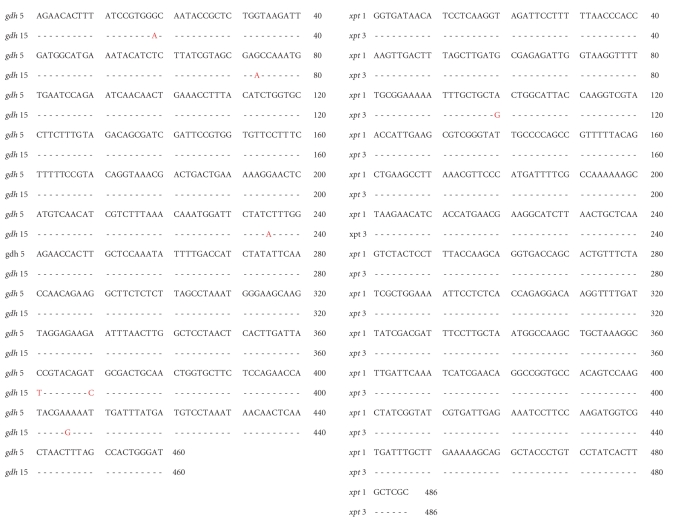
Alignment of sequences for alleles *xpt* and *gdh* for ST1989 and ST180 demonstrating one SNP difference in the *xpt* gene at position 100 and six SNP differences in the *gdh* gene at positions 19, 73, 235, 361, 370, and 406.
